# Electrical and Physical Characteristics of WO_3_/Ag/WO_3_ Sandwich Structure Fabricated with Magnetic-Control Sputtering Metrology †

**DOI:** 10.3390/s18092803

**Published:** 2018-08-25

**Authors:** Shea-Jue Wang, Mu-Chun Wang, Shih-Fan Chen, Yu-Hsiang Li, Tien-Szu Shen, Hui-Yun Bor, Chao-Nan Wei

**Affiliations:** 1 Department of Materials and Resources Engineering, National Taipei University of Technology, Taipei 10608, Taiwan; sjwang@ntut.edu.tw (S.-J.W.); macsfchen@gmail.com (S.-F.C.); samli0316@yahoo.com.tw (Y.-H.L.); 2 Department of Electronic Engineering, Minghsin University of Science and Technology, Hsinchu 30401, Taiwan; tss@must.edu.tw; 3 Chung-Shan Institute of Science and Technology, Taoyuan 32546, Taiwan; borhy.h12@msa.hinet.net (H.-Y.B.); chaonien@ms41.hinet.net (C.-N.W.)

**Keywords:** sputtering, transparent conductive film, tungsten oxide, sensing, current-voltage characteristics, Schottky contact

## Abstract

In this work, three layers of transparent conductive films of WO_3_/Ag/WO_3_ (WAW) were deposited on a glass substrate by radio frequency (RF) magnetron sputtering. The thicknesses of WO_3_ (around 50~60 nm) and Ag (10~20 nm) films were mainly the changeable factors to achieve the optimal transparent conductivity attempting to replace the indium tin oxide (ITO) in cost consideration. The prepared films were cardinally subjected to physical and electrical characteristic analyses by means of X-ray diffraction analysis (XRD), field-emission scanning electron microscope (FE-SEM), and Keithley 4200 semiconductor parameter analyzer. The experimental results show as the thickness of the Ag layer increases from 10 nm to 20 nm, the resistance becomes smaller. While the thickness of the WO_3_ layer increases from 50 nm to 60 nm, its electrical resistance becomes larger.

## 1. Introduction

The transparent conductive film (TCF) offers versatile and cost-effective applications with a light transmittance of 80% or more in visible light range (400 to 800 nm) and low resistivity [1], sometimes treated as optical or electrical sensors [2,3,4]. With the rapid expansion of the market for photovoltaic elements, the demand is rapidly increased, so is the pursuit of high stability and low cost processes in production. The transparent conductive film should exhibit both transparent and conductive properties. Currently, most of the materials are metal oxides, called transparent conducting oxides (TCO), especially with indium tin oxide (ITO) [5]. It was found that ITO material can be employed as an ohmic contact layer [6]. However, the “indium” element in indium tin oxide is a rare metal and expensive, so that many manufacturers and research institutes in the world are proactively developing the ITO-like materials to replace the previous. It was found that if a metal layer was added to the conductive film to form a multilayer film structure, the overall conductivity and mechanical properties of the film were possibly and greatly increased instead of ITO. Depositing a suitable metal thickness in a clamping oxide structure seems possible in the recent development stage, without affecting the visible light transmittance while still maintaining its low-resistance properties. This is also the aim of this work; to make an acceptable cost-effect TCF in future applications.

Most of metal thin films such as Au, Ag, Cu, Pt, and Al are, basically, good infrared reflectors. If the optical transparency in the tunable region is strongly enhanced, the preferred thickness of the metal film layer is less than 10–20 nm. Theoretically, the metal thin film can be treated as a good transparent conductive film, but when the thickness of the metal thin film is less than 10 nm, it is easy to form discontinuous island-like films, so that the resistivity of the thin film increases and the scattering incidence light in turn reduces the transparency of the film itself [1]. Metal oxide films have a long history of application to TCO, and cadmium oxide (CdO) was first prepared by sputtering in 1907 by Badeker. In 1954, Rupprecht vapor-deposited metal indium (In) onto a quartz substrate and oxidized it at 700 to 1000 °C in an atmosphere (oxygen: nitrogen = 1:4) to obtain transparent and conductive indium oxide (In_2_O_3_). Furthermore, it is inferred that the conductivity of this film is a crystal defect derived from the film structure itself. In 1968, Philip found that the doping of Sn ions in the indium oxide film enhanced the conductivity of the overall film, and then produced In_2_O_3_:Sn film [7], now widely consolidated in indium tin oxide [8,9,10] applications.

The transparent conductive oxide of the metal is transparent in the visible region. For electrical properties, these oxides are usually doped with some metal impurities, doped with metal ions that are more than one cation of the original compound to form an n-type semiconductor, or doped with a non-metal ions less than its anion to form a p-type semiconductor. It is also probable to generate a compound having an incomplete oxidation state, so that an anion void is contained in the material, and WO_3_ according to the present experiment is usually an n-type semiconductor material and has a work function (Φ_s_) around 5.7 V.

Among many metal materials, silver (Ag) material is a good choice in conductivity and relatively good photoelectric characteristics. Accompanying tungsten oxide (WO_3_), a semiconductor material with a large energy gap, it has a chance to form a transparent conductive film mainly composed of tungsten oxide and a silver metal layer in the middle. According to different characteristics of the WO_3_/Ag/WO_3_ transparent conductive film, it is expected to develop different application components and products in the future. Tungsten oxide (WO_3_) generally proposes a molecular weight of 231.838 g/mol, a density of 7.2 g/cm^3^, and a melting point of about 1473 °C and is soluble in water and ammonia, but slightly soluble in hydrofluoric acid, and, however, insoluble in acid. Tungsten compounds can usually exhibit five different ionization states, W^2+^, W^3+^, W^4+^, W^5+^, and W^6+^. There are also cases where codons of different valences coexist. WO_3_ is the highest valence oxide of tungsten. As usual, the oxygen content in tungsten oxide does not exactly meet the full stoichiometric ratio of O:W = 3:1, so that the chemical formula is often expressed as WO_3−x_. The conductive mechanism of the tungsten oxide is chiefly via oxygen vacancies. Oxygen vacancies are mainly formed because of the large number of tungsten oxides arranged, and the oxygen atoms in the structure are vacant due to defects in the material itself. In this case, the chemical formula of tungsten oxide should be WO_3−x_, and the sporadic oxygen vacancies will generate a new energy level below the conduction band. At the generated energy level, the active electrons are able to be transited to the upper energy levels. The conduction band generates free electrons and is thus electrically conductive [5].

**Figure 1:**
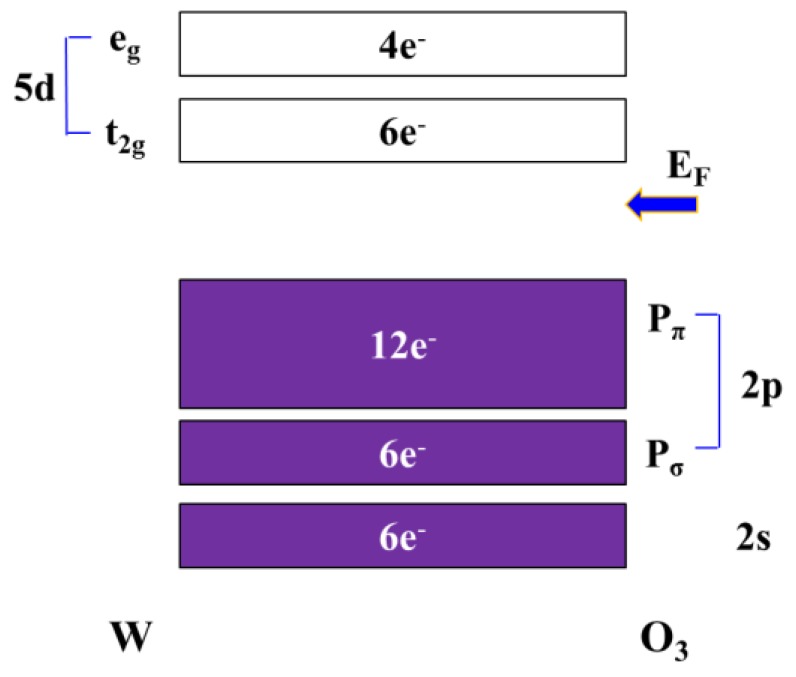
Simple sketch of band diagram of WO_3_, where E_F_: Fermi level in the location. Filled energy states are colored.

**Figure 2:**
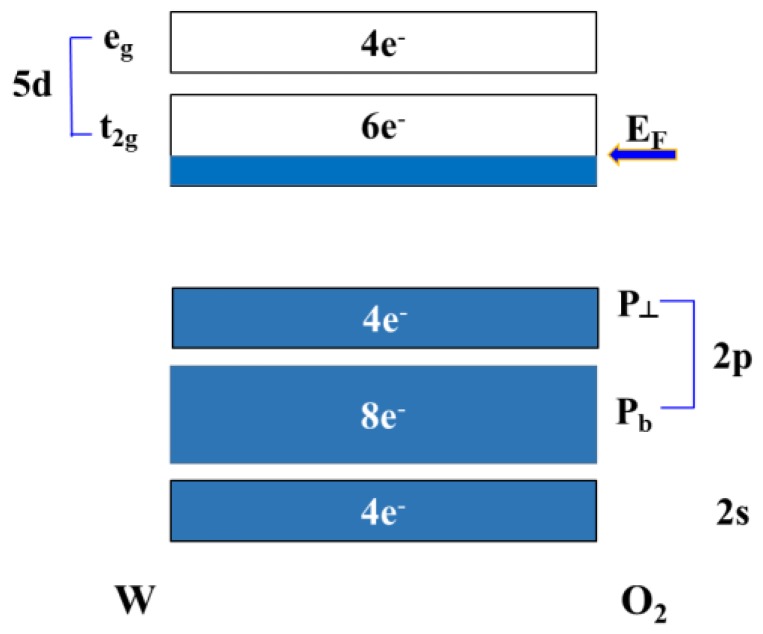
Schematic band profile of WO_2_, where E_F_: Fermi level in the location. Filled energy states are colored.

As shown in Figure 1, the valence band of WO_3_ is composed of O atoms 2s, 2p orbitals, and the conduction band is composed of W atoms 5d orbitals. According to the crystal field theory, the 5d orbital division of W is separated into two parts, e_g_ and t_2g_, but they are all unfilled orbits. Therefore, the WO_3_ valence band is cardinally localized in the O(2p) orbital, the conduction band is localized in the t_2g_ orbital of the central metal W^6+^, and the Fermi level is between the O(2p) band and the W(t_2g_) band [8,11,12,13,14]. The other possible formation of tungsten oxide, as shown in Figure 2, is WO_2_. The Fermi level (E_F_) of WO_2_ is generally in the conduction band, so that active electrons do not need much energy to look for the quantum state of the vacancy for jumping and inducing current conduction. The bandgap of WO_2_ is not remarkably observed.

## 2. Fabrication of WAW Conductive Films

The chosen substrate in this work is a glass substrate, which is a level of sodium glass. The selected target of tungsten is a 2-inch circle type with the integrity 4N. In the experiment, the flowing gases are pure argon and oxygen. Before the sputtering, the glass substrate must be cleaned well with acetone, de-ionized (DI) water, and alcohol in the operation of ultra-sonic equipment. After that, the de-dry process with nitrogen is subsequently executed. Before the beginning of sputtering, the glass substrate required for the experiment must be cleaned, placed in a beaker containing acetone, deionized water, and alcohol, and then in an ultrasonic oscillator for 10 min. The function of acetone is to remove the organic matters on the surface of the substrate. Deionized water can take out the dust and chemicals on the substrate surface. Alcohol takes the surface moisture of the substrate away. After cleaning, the residual alcohol on the surface of the glass substrate should be fully dried out by a nitrogen gun.

### 2.1. WAW Film without Plated Electrode

**Figure 3:**
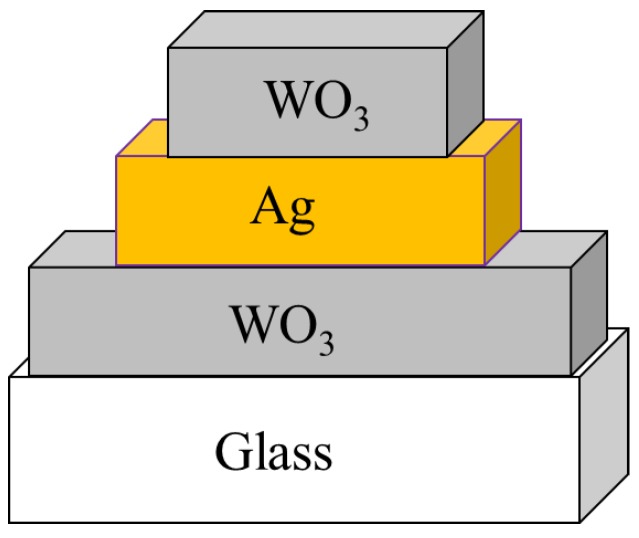
Three-layer transparent conductive film structure of WO_3_/Ag/WO_3_ without metal electrodes.

**Figure 4:**
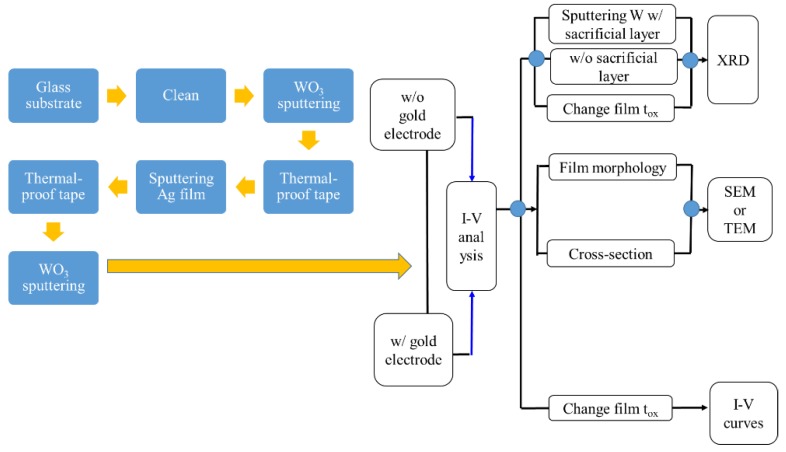
Schematic chart of process flow to fabricate a WAW film without (w/o) or with (w/) gold electrodes.

**Figure 5:**
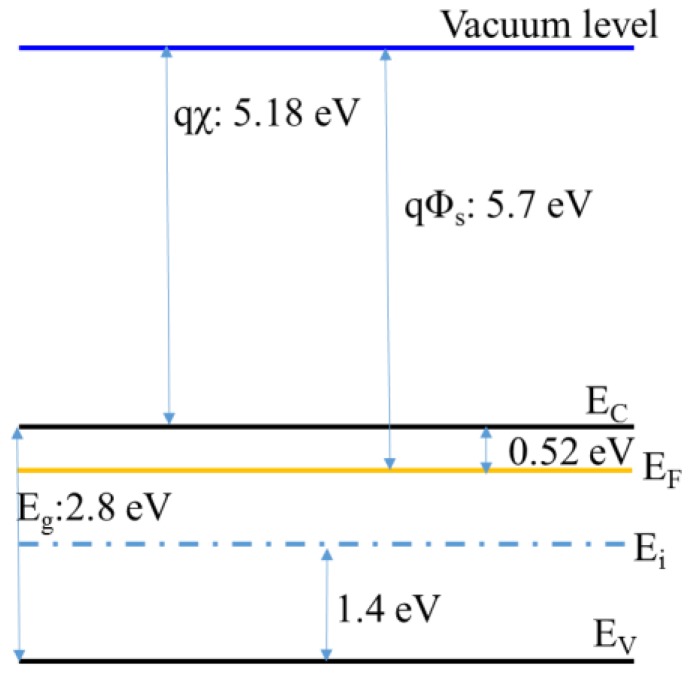
Schematic diagram of band diagram of WO_3_ film as an n-type semiconductor.

After the cleaned substrate was placed in the sputtering chamber, the magnetron sputtering system was step by step turned on for sputtering, following the standard operation procedures, and the mechanical pump was used to pump the chamber. When the pressure of the chamber reached 0.05 torr, the diffusion pump was added and pumped up to the background pressure of 2 × 10^–5^ torr for tungsten oxide deposition the gas introduced into the chamber was argon (flow rate: 30 sccm) and oxygen (flow rate: 24 sccm), and the working pressure was 7 × 10^–3^ torr. For deposition of a silver layer, the gas was argon (flow rate: 30 sccm), and the working pressure was 6 × 10^–3^ torr, and the tunable rotation speed was set to 300 rpm. Subsequently, the metal target must be pre-sputtered for 15 min before the start of sputtering to remove impurities from the target surface. After the plasma environment stabilized, the baffle between the target and the stage was slowly removed, and the procedure of film deposition was begun. Continuously, after the sputtering completed, the test piece was taken out. Ultimately, the plating analysis and the property test for the deposited films were performed. During the sputtering process, the thicknesses of the silver (10 or 20 nm) and the tungsten oxide (50 or 60 nm) and the RF power to generate the plasma were the main changeable factors, as shown in Table 1. Pre-sputtering the surface of the target for 15 min was to remove the surface impurities and oxide layer to clean the target surface well. After the pre-sputtering, removing the baffle and sputtering three layers of WO_3_/Ag/WO_3_ film, as shown in Figure 3, were executed in sequence. After sputtering each layer of film, a layer of heat-resistant tape defined the deposition, or played a similar function of photolithography and etching in the semiconductor manufacturing to define a desired pattern. Because the upper film areas are smaller than the underlying film, this three-layer film stacked was like a pyramid. The physical and electrical properties of the entire WAW films were sensed and verified with an X-ray diffraction analyzer (XRD), a field emission electron microscope (FE-SEM), a UV/Vis ultraviolet/visible spectrometer, and a Keithley 4200 semiconductor parameter analyzer. The simple process flow is demonstrated in Figure 4. The schematic energy diagram of WO_3_ film is represented in Figure 5. The work function of WO_2_ is in the range from 4.41 to 4.6 V.

### 2.2. WAW Film Coated with Metal Electrodes

**Figure 6:**
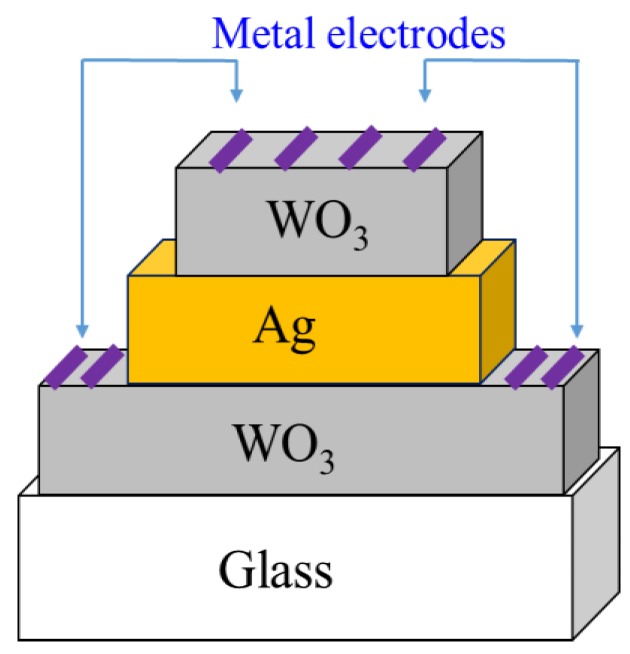
Three-layer transparent conductive film structure of WO_3_/Ag/WO_3_ with deposited metal electrodes.

In order to probe how adding electrodes influences the electrical characteristics of WAW films, the metal electrodes providing the different work functions, as shown in Table 2 [15,16,17], were deposited and patterned as a comparison group. In this formation of WAW films, except for the metal sputtering as electrodes, the sputtering method was the same as above and the changing thicknesses of the silver (10 or 20 nm) and the tungsten oxide (50 or 60 nm) were also the same. After that, a layer of heat-resistant tape was also applied to the WAW film to control the deposition area to form the desired patterns. Furthermore, the area of the heat-resistant tape was used to control the area of each film. Consequently, pyramid WAW films with three different patterned electrodes of Au, Ag, or Cu electrodes with 10nm thickness sputtered on the tungsten oxide layer were formed, as shown in Figure 6, to provide the Kevin structures in the electrical performance, exposing the ohmic, space-charge limited (SCL), or trap-filling limited (TFL) mechanisms [18,19,20]. The areas of each metal electrode for upper and bottom tungsten-oxide films are 4 mm × 1 mm and 12.5 mm × 1 mm, respectively. The areas of each layer for WAW film are 25 mm × 12.5 mm, 15 mm × 7.5 mm, and 10 mm × 4 mm from bottom to top, respectively. The physical and electrical characteristics of this WAW film with sputtered electrodes were also measured and analyzed by using an X-ray diffraction analyzer (XRD), a field emission electron microscope (FE-SEM), and a Keithley 4200 semiconductor parameter analyzer.

## 3. Measurement Results and Discussion

In the measurement results, we discuss the electrical and physical performance of the WAW film stack. The optical transparency of the WAW film at wavelength = 550 nm is slightly investigated.

### 3.1. Microstructure Analysis of Silver Film Surface

**Figure 7:**
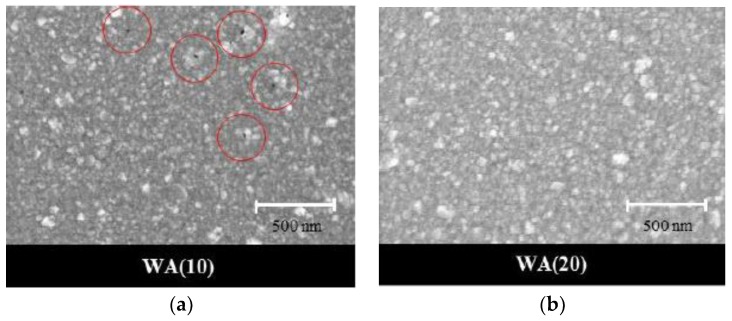
Scanning electron microscope (SEM) images of WA films at (**a**) 10-nm Ag (A10) film and (**b**) 20-nm Ag (A20) film.

First of all, the bottom WO_3_ with 50-nm thickness was deposited. Then, the silver (Ag) layer was sputtered up to 10 nm. During this time, the surface of this Ag film demonstrated fine black holes, which means that the Ag film was not sufficiently thick to form a continuous film. Nevertheless, as the thickness of the silver was increased up to 20 nm, the pores completely disappeared and the silver film became uniform, as shown in Figure 7.

### 3.2. X-ray Diffraction (XRD) Analysis

**Figure 8:**
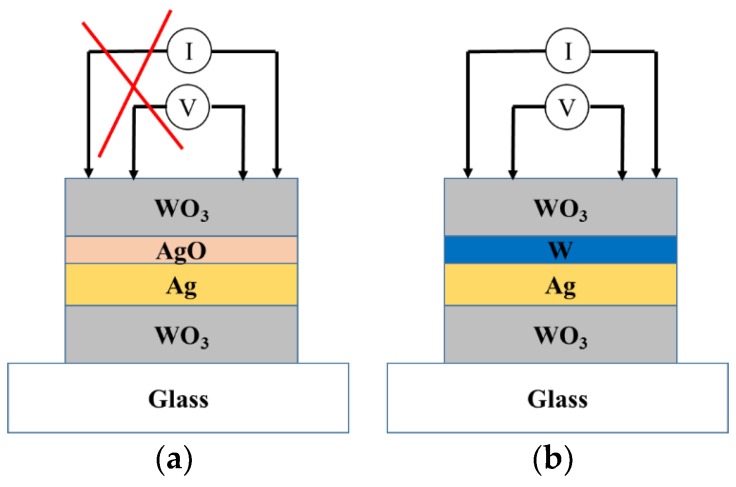
Schematic diagram of plating: (**a**) without barrier layer W on Ag film and (**b**) with W layer on Ag film.

**Figure 9:**
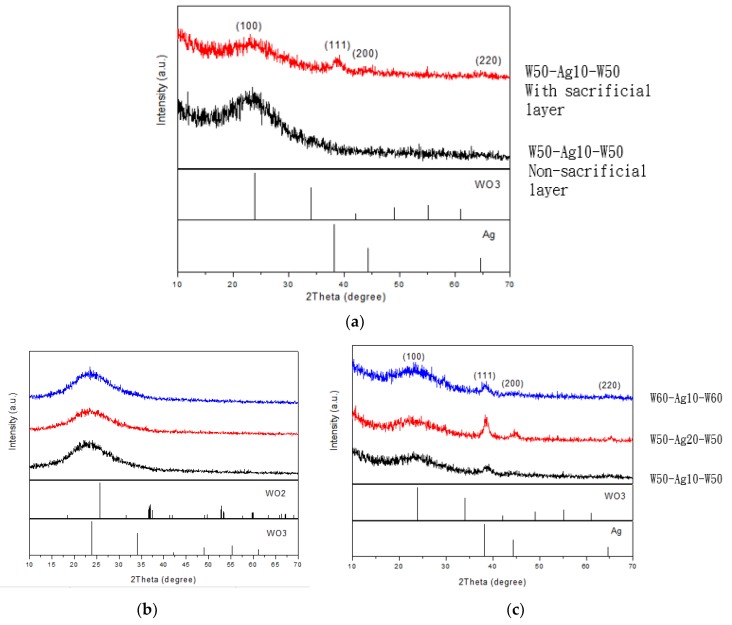
X-ray diffraction (XRD) patterns: (**a**) the WAW (W50: W50 nm/Ag10: Ag10 nm/W50: W50 nm) film with or without a sacrificial layer; (**b**) WO_3_ (50nm) film sensed with three locations; and (**c**) different film thicknesses of WAW films.

As WO_3_/Ag/WO_3_ (WAW) sample was coated without a sacrificial layer W before sputtering the upper layer of tungsten oxide, the Ag film was easily oxidized as silver oxidation (AgO) decreasing the conductivity, which is not desired in applications. To prevent Ag film from the oxidation depicted in Figure 8a, a barrier layer (or called sacrificial layer) W (5-nm thickness, labeled as W5) was deposited after the formation of Ag film immediately, as shown in Figure 8b. The silver oxide [21,22,23] formed decreases the electrical conduction of the silver layer in the WAW structure and no XRD peak of silver film observed definitely represents the silver film in this un-remarkable crystallization, shown in Figure 9a. The intensity of the silver layer with XRD pattern shown in Figure 9a correlated to Figure 8b exhibits (111), (200), and (220) orientation peaks. The possible reason is that during the process of depositing the coating layer, the oxygen of the reaction gas flowing into the chamber was dissociated into oxygen ions by the plasma, and the silver was easily combined with oxygen, yet the active oxygen ions were made before the deposition of the tungsten oxide. 

In the XRD pattern analysis, the WO_3_ (50 nm) and Ag (10 nm) films were measured separately. It can be found that the diffraction peak of tungsten oxide was not observed in Figure 9b, and the broad diffraction peak of 2θ at 20°~30° indicated that the buffer layer tungsten oxide was amorphous. Since the WO_3_ film formed by sputtering with the W target during sputtering, to verify whether there was oxidative unevenness, three XRD points at different locations were measured. The XRD characteristics expressed the same, and all of them were amorphous structures. In Figure 9c, in the WAW films, the thickness of the silver film was increased, and the XRD diffraction peak was apparently observed. It was found that as the thickness of the silver film increased, the intensity of the diffraction peak also increased, indicating an increment in crystallinity.

### 3.3. Current-Voltage Characteristics Analysis

**Figure 10:**
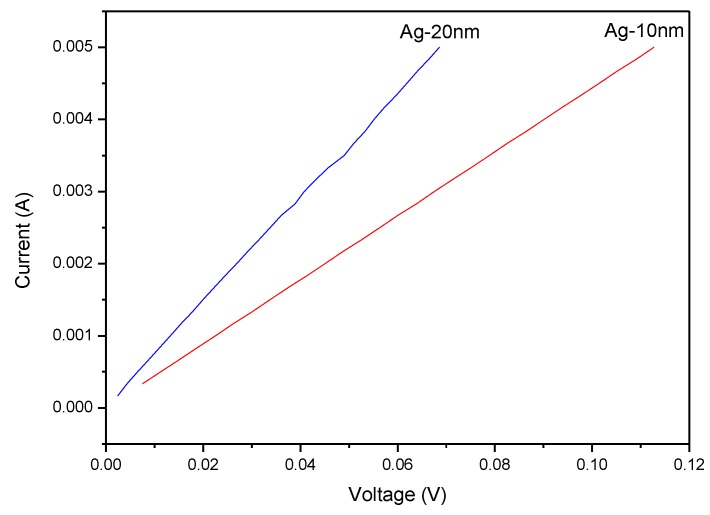
Current-voltage characteristics of single-layer Ag film.

The quality of Ag films on glass substrate, besides the observation with SEM photos in the material view, also can be evaluated with I–V characteristics, as shown in Figure 10 representing 10- and 20-nm Ag films. These two consequences are consistent and demonstrate that the thickness of Ag film increased and the resistance of that decreased.

**Figure 11:**
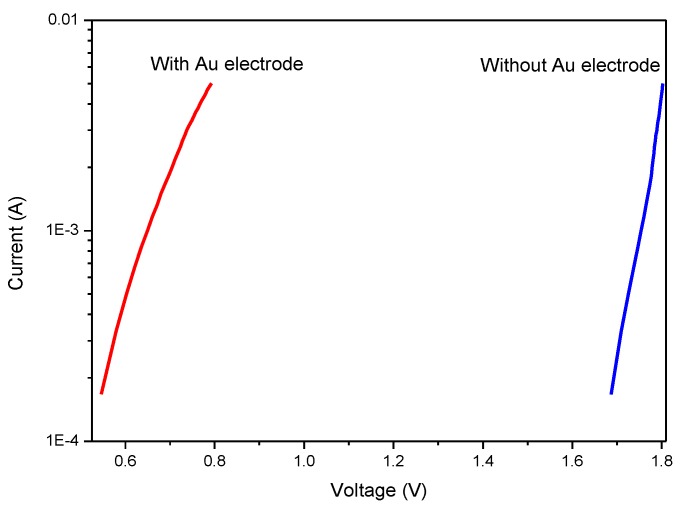
I–V curves of single layer WO_3_ with or without gold electrode.

If the WAW film is deposited with a metal electrode such as gold metal, the voltage drop at the starting voltage for a single WO_3_ film is tremendously improved, as shown in Figure 11. The material of the probe needle, or tungsten-titanium needle, indeed affects the electrical performance due to the probing contact resistance. While the current is fixed at 1 mA, the voltage difference between both cases is about 1.1 V from 0.65 to 1.75 V. The measurement error due to the probe contact can be reduced, which is the main benefit with the sputtering process of a gold electrode.

### 3.4. Light Transmittance Analysis

#### 3.4.1. Optical Transmittance of WA Film

**Figure 12:**
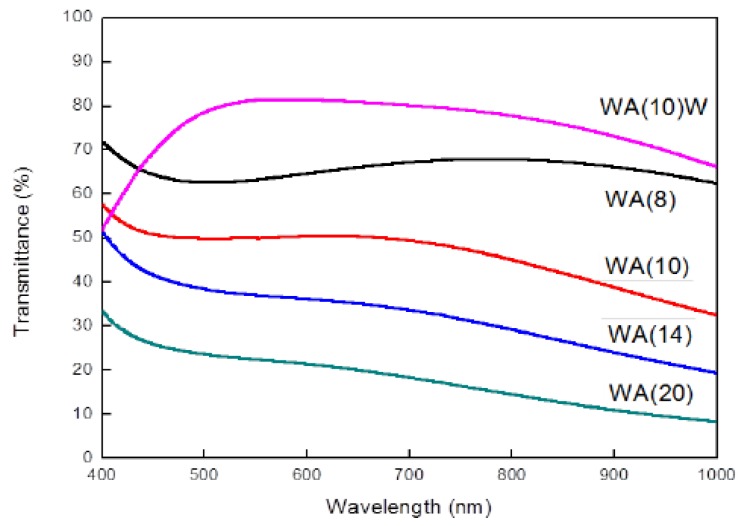
UV-Vis spectrum of WA film at different silver thicknesses as the bottom WO_3_ thickness fixed.

According to the UV-Vis spectrum of WA films, as shown in Figure 12, we observe that the optical transmittance (T %) [24,25] is not uniform or regular with the visible wavelengths [26]. If the top WO_3_ layer treated as an anti-reflective layer was deposited with 50 nm thickness, the transmittance in WA(10)W could be increased. If there were only two layers, bottom WO_3_ with fixed 50 nm thickness and silver layer, the thickness of the silver layer was increased and the transmittance performance was reduced. Reversely, the conductivity of the silver layer was increased according to the previous analysis. Using the wavelength = 550 nm as an example, the transmittance with the variables of silver thickness is represented at Table 3. The overall visible light transmittance at WA(8) group on average is more than 60%.

#### 3.4.2. Optical Transmittance of WAW Film

**Figure 13:**
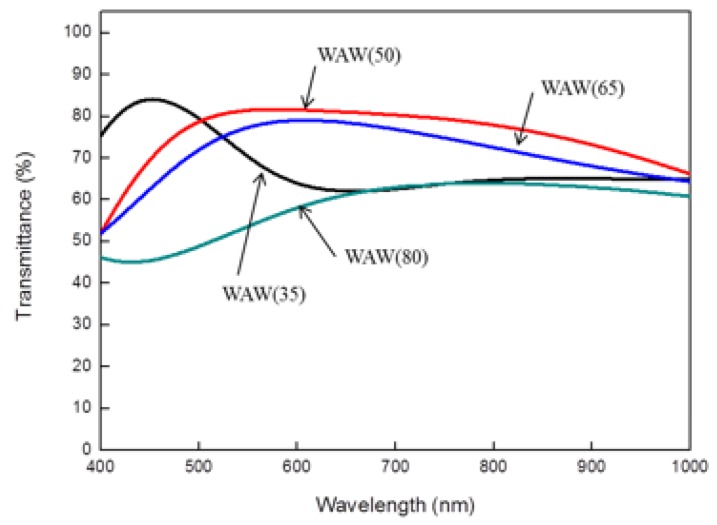
UV-Vis spectrum of WAW films at different top-WO_3_ thicknesses.

Understanding the transmittance versus the top WO_3_ thicknesses as an anti-reflective layer is also important for the transparent conductive film. Following the previous section, the manufacturing process for the bottom WO_3_ (W(50)) and silver layers (A(10)) was identical, but the thicknesses of the top WO_3_ layer were changeable. The measurement results with UV-Vis spectrum are shown in Figure 13. The anti-reflectivity at the WAW(50) group is the best, which means the transmittance at = 550 nm is the maximum in these four groups, as shown Table 4 [27].

## 4. Conclusions

Transparent conductive films (TCF) [28] have a wide range of applications and a worldwide market. This benefit attracts the researchers to seek some feasible metrology to replace the rare metal, such as indium in ITO applications. Considering and balancing the electrical conductivity, optical transmittance, and cost effect in TCF manufacturing is zealously desired. Using a magnetron sputtering technology [29,30] different from electron beam evaporation [31] to make WAW film instead of ITO film [32] is indeed successful in this work. 

Following the analysis of XRD results and SEM images to examine the crystallinity of silver film or tungsten oxide can provide a suitable growth thickness of the silver layer or tungsten oxide. With the electrical measurement and optical spectrum scanned, the optimal sputtering parameters in thickness control of the top and bottom of the tungsten oxide and silver layer can be wholly extracted and confirmed. The tunable thickness of the top tungsten oxide as an anti-reflective layer is intensely related to the transmittance. Summarizing these sensed consequences, the optimal thicknesses of WAW film should be W(50)/A(20)/W(50) in this experiment.

In this work, a thin barrier layer of tungsten on the silver film was necessarily deposited to prevent this film from oxidation and keep great conductivity for the full WAW film. In the electrical measurement, if the metal electrode is deposited, the probe resistance between the tungsten oxide and probe needle can be diminished more and the parasitic resistance confusing the measurement error also declined.

## Figures and Tables

**Table 1:** Changeable sputtering parameters in this work.

Sputtering Method	Sputtering Power (W)	Thickness of Tungsten Oxide (nm)	Thickness of Silver Layer (nm)
RF type	100	50 or 60	10 or 20

**Table 2:** Work functions (Φ_m_) for several useful metals.

Metal Species	Au	Ag	Cu	W
Work function (Φ_m_, V)	4.8	4.26	4.65	4.55

**Table 3:** Light transmittance of WA film under various silver thicknesses.

Sample	WA (8 nm)	WA (10 nm)	WA (14 nm)	WA (20 nm)	WA (10 nm)W
T % at = 550 nm	63.28%	50.06%	37.09%	22.49%	81.34%

**Table 4:** Light transmittance of WAW films at different top-WO_3_ thicknesses.

Sample	WAW(35)	WAW(50)	WAW(65)	WAW(80)
T % at = 550 nm	70.37%	81.34%	77.28%	53.58%
